# An individually randomized controlled trial of a mother-daughter HIV/STI prevention program for adolescent girls and young women in South Africa: IMARA-SA study protocol

**DOI:** 10.1186/s12889-021-11727-3

**Published:** 2021-09-20

**Authors:** Geri R. Donenberg, Millicent Atujuna, Katherine G. Merrill, Erin Emerson, Sheily Ndwayana, Dara Blachman-Demner, Linda Gail Bekker

**Affiliations:** 1 Center for Dissemination and Implementation Science, Department of Medicine, University of Illinois at Chicago, 818 S. Wolcott, Chicago, IL 60612 USA; 2 Desmond Tutu HIV Center, Cape Town, South Africa; 3 Office of Behavioral and Social Sciences Research, National Institutes of Health, Bethesda, MD USA

**Keywords:** HIV prevention, Adolescent girls and women, South Africa, Mother-daughter intervention

## Abstract

**Background:**

South Africa has the world’s largest HIV epidemic, but South African adolescent girls and young women (AGYW) acquire HIV at twice the rate of and seroconvert on average 5–7 years earlier than their male peers. Female caregivers (FC) are an untapped resource for HIV/STI prevention in South Africa and offer a novel opportunity to strengthen AGYW prevention efforts. This study will evaluate the effectiveness and cost-effectiveness of an evidence-based mother-daughter HIV/STI prevention program tested in the United States and adapted for South Africa, Informed Motivated Aware and Responsible Adolescents and Adults (IMARA), to decrease STI incident infections and increase HIV testing and counseling (HTC) and PrEP uptake in AGYW.

**Methods:**

This is a 2-arm individually randomized controlled trial comparing IMARA to a family-based control program matched in time and intensity with 525 15–19-year-old Black South African AGYW and their FC-dyads in Cape Town’s informal communities. AGYW will complete baseline, 6-, and 12-month assessments. Following randomization, AGYW-FC dyads will participate in a 2-day group workshop (total 10 h) that includes joint and separate mother and daughter activities. Primary outcomes are AGYW STI incidence, HTC uptake, and PrEP uptake at 6 months. Secondary outcomes are AGYW STI incidence, HTC uptake, and PrEP uptake at 12 months, sexual behavior (e.g., condom use, number of partners), HIV incidence, and ART/PrEP adherence and intervention cost-effectiveness. AGYW who test positive for a STI will receive free treatment at the study site. HIV positive participants will be referred to ART clinics.

**Discussion:**

Primary prevention remains the most viable strategy to stem new STI and HIV transmissions. HIV and STI disparities go beyond individual level factors, and prevention packages that include supportive relationships (e.g., FC) may produce greater reductions in HIV-risk, improve HTC and PrEP uptake, and increase linkage, retention, and adherence to care. Reducing new HIV and STI infections among South African AGYW is global public health priority.

**Trial registration:**

ClinicalTrials.gov Number NCT04758390, accepted 02/16/2021.

## Background

South Africa (SA) is home to the largest number of people living with HIV worldwide, [1, 2] but within-population differences exist [2–4]. HIV rates are higher among women than men, [2, 3] adolescent girls and young women (AGYW) acquire HIV at twice the rate and seroconvert on average 5–7 years earlier than male peers, [2] and AIDS is the second leading cause of death among adolescents [5]. Moreover, AGYW account for over 67% of new HIV infections, [6] but only 15% know their status [7]. Women’s HIV disparities are explained by a multiplicity of social and structural inequities that shape and constrain HIV-risk behaviors and continue to drive incident infections in SA [8–13]. Hence, multilevel prevention packages that are tailored to the local epidemiology and cultural context are likely to achieve and sustain maximum reductions in AGYW HIV-risk [14–21].

Families are a relatively untapped resource in SA’s HIV prevention efforts. Yet, families are uniquely positioned to support prevention, because they are the first socialization experience for adolescent sexual and reproductive health. Mothers and other important female caregivers (FC) (e.g., aunts, cousins) play a central role in AGYW’s sexual behavior and development, and can be effective partners in reducing sexual risk while promoting new biomedical technologies like PrEP and HIV testing and counseling (HTC) [22, 23]. Research that optimizes uptake and adherence to PrEP and HTC in women [24] will facilitate more equitable access to all HIV scientific advances. Research in the US indicates maternal warmth and attachment, close family relationships, parental involvement and support, and positive parenting predict consistent condom use, less exposure to HIV-risk situations, and later sexual debut among adolescents [25–29]. Likewise, mother-daughter communication that is open, receptive, and comfortable is associated with less risky sexual behavior in the US [26, 30–37] and in Africa [38–43].

South African FC may serve as a catalyst for AGYW change. Despite noting parents as the best source of information about sex and general receptivity to such discussions, only 20% of SA youth receive information about sex from their parents, and almost 50% of girls report that they could not openly discuss sexuality with their parents [44, 45]. Likewise, African mothers report needing and wanting accurate knowledge to discuss sex with their children [46]. Capitalizing on the FC-AGYW relationship, improving the quality of FC-AGYW conversations about sexual and reproductive health, teaching FC to use accurate information, and challenging cultural taboos that inhibit communication about sex [47] may alter the current HIV-trajectory for SA AGYW. Notably, AGYW may also be change agents for their FC who want to be positive role models for AGYW. In the process of promoting AGYW sexual and reproductive health, FC may change their own behavior (including HTC and PrEP uptake), thereby reducing their own risk for HIV.

IMARA is an evidence-supported mother-daughter (“mother” refers to any female caregiver) program based on the Social Personal framework [48] and derived from three established interventions: SISTA [49–53] and SiHLE [54] for African American women and girls respectively, and Project STYLE for teens and families in mental health care [55]. SISTA and SiHLE address the importance of relationship power and gender dynamics as drivers of HIV risk behavior. This is critical for the South African context where AGYW’s first and ongoing sexual partner is typically an older male, [56] and AGYW with older partners are four times more likely to test positive for a sexually-transmitted infection (STI), [57] report transactional sex, [57] and have high HIV infection rates [58]. In addition, gender-based violence (GBV) among SA AGYW is higher than in every other region in sub-Saharan Africa, [44, 59, 60] and is related to high rates of STI [61] and HIV-risk, [62–64] and impedes women’s ability to receive HTC, link to treatment, and adhere to PrEP [65–67]. Project STYLE emphasizes parent-teen relationships and communication as protective against HIV and STI risk. A recent efficacy study of IMARA in the US [68] revealed a 43% reduction in incident STI infections at 1-year follow-up among 14–18 year-old African American girls who received IMARA compared to a time-matched health promotion program (*p* = .011).

Adaptation of IMARA to the South African context is important to ensure acceptability and uptake. Added content related to HTC and PrEP, two essential strategies to comprehensive HIV prevention, [69–74] will equip AGYW and FC with greater control and agency over their sexual lives [75]. Moreover, IMARA could provide a novel approach to reduce AGYW STI and HIV-risk and improve HTC and PrEP awareness, acceptability, and uptake (where appropriate) for FC and AGYW. Finally, rigorous cost evaluations of HIV and STI prevention packages are sorely needed to optimize the use of available resources. Establishing the cost-effectiveness (CE) of HIV prevention packages is essential if they are to be sustained [76, 77].

In sum, SA AGYW have not benefitted equally from gains in the HIV epidemic. The unrelenting disparity of new infections in this group [78] requires an expansion of prevention options within the SA context [79]. Focusing on vulnerable populations and high-incidence locations, i.e., SA AGYW, will realize the greatest benefits in altering epidemiological trajectories [80]. While changing behavior can reduce HIV and STI acquisition, [11] research on evidence-based interventions with strong evaluation designs is lacking for AGYW, especially those capitalizing on the strengths and assets of families.

### Study objectives

The purpose of this study is to test the effectiveness and cost-effectiveness of a culturally-tailored, evidence-based, HIV/STI prevention program for SA AGYW and FC. We will randomly assign 525 AGYW-FC dyads to IMARA-SA (*n* = 263) or a family-based health promotion (HP) program (*n* = 262) matched in time and intensity and compare participants on incident STI, and HTC and PrEP uptake at 6 months. Secondary objectives are to assess intervention effects on the primary outcomes at 12 months, reported non-condom use, number of partners, HIV incidence, and adherence to ART/PrEP. We will also evaluate the costs and CE of IMARA with respect to the acquisition of STI and HIV where possible, considering power limitations.

## Methods

### Design

This is an individually randomized controlled trial (RCT) of IMARA for 15–19 year-old Black South African AGYW and their FC. AGYW-FC dyads will be randomized to IMARA-SA versus a family-based HP program. All participants will complete baseline, 6-, and 12-month follow-up assessments via audio computerized assisted self-interview technology (ACASI) and provide urine to screen for three sexually transmitted infections (STI). All participants will also be offered HTC and PrEP, and where appropriate (e.g., if HIV/STI positive), will receive free care at the on-site adolescent-friendly reproductive health clinic at the Desmond Tutu Health Foundation (DTHF) and referral to public clinics.

### Identification and recruitment of participants

**Fig. 1:**
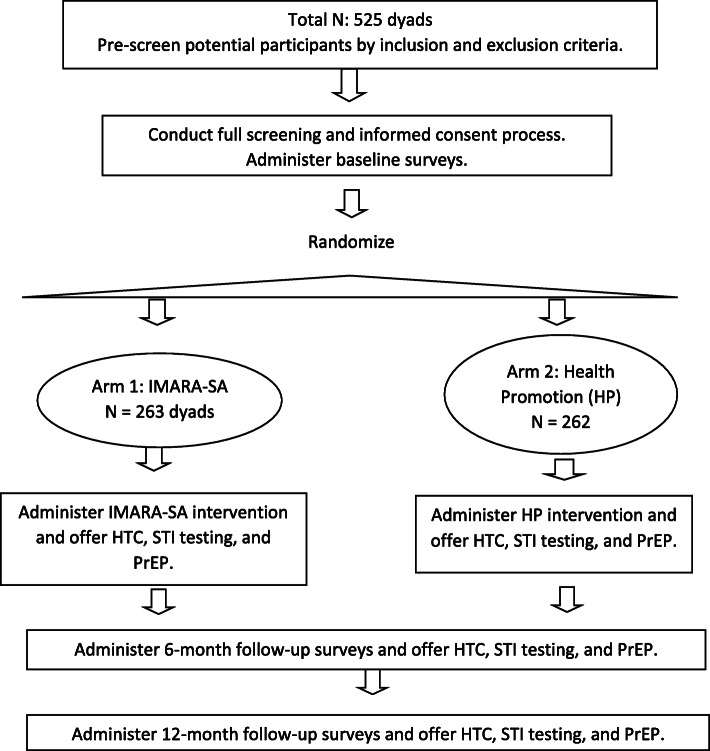
Schema for an individually-randomized controlled trial of the IMARA-SA intervention

The study will take place in Phillippi and the surrounding townships including Nyanga, Gugulethu and Khayelitsha, located in the Cape Town metropolitan. These areas are densely populated, have a high HIV burden, and are poorly resourced. Recruitment strategies include street outreach, word-of-mouth referrals, flyers, and clinic-based contacts by study personnel. The majority of the residents are Black and mixed race. We will enroll AGYW and a FC, defined as an important and influential female in AGYW’s life (e.g., mother, aunt). AGYW will be: a) Black or mixed race; b) 15–19 years-old; c) residing in Phillipi and the surrounding areas who d) speak Xhosa, English, or a combination, as these are the primary regional languages. AGYW may or may not be sexually active, and information about AGYW sexual activity will not be shared with FC. FC will be: a) selected by the AGYW and agreed upon by the legal guardian; b) 24 years or older; c) living with or in daily contact with the AGYW; and d) a speaker of Xhosa and/or English. We will include HIV-infected and uninfected FC and AGYW, and AGYW or FC who become HIV+ and/or pregnant during the study may continue to participate. AGYW and FC must agree to participate as a dyad, but AGYW refusal will supersede FC consent. All participants, regardless of randomization status, will be followed for 12 months with visits conducted at 6 and 12-month intervals (Fig. 1).

### Randomization

We will use a 1:1 randomization allocation to assign FC-AGYW dyads to treatment arm (IMARA-SA *n* = 263 or HP *n* = 262) following the baseline assessment. The AGYW will select a colored paper from a hat that determines the family’s group assignment. Assessors but not intervention staff will be blinded to treatment arm at 6- and 12-month follow-up.

### Training interventionists (IMARA-SA and health promotion)

Training for both conditions will last approximately 30 h, and we will train IMARA-SA and HP interventionists separately. Interventionists will review their assigned curriculum and related training manuals. Training for both programs will stress the importance of following the manualized protocol to ensure fidelity across interventionists and of completing fidelity measurements at the end of each workshop day. Following review of the curriculum, interventionists will practice delivering each activity, taking turns serving as group leaders and participants. Each interventionist will be “certified” as competent to deliver IMARA-SA or the HP program by the site Principal Investigator and study sub-investigator. Refresher trainings will occur annually and as needed if observer fidelity ratings indicate drift and non-compliance.

### Supervision, quality assurance, and treatment Fidelity

We will use a detailed manual and facilitator guide, conduct weekly supervision, and assess fidelity using facilitator self-report and observer ratings of adherence and competence (see below). Adherence measures will determine if the program was delivered as intended (yes/no), and competence ratings will indicate the quality of delivery. During ongoing supervision, a trained member of the study team will review fidelity reports, discuss session activities, problem-solve difficulties, and provide interventionists with feedback. If interventionists deviate from the curriculum, the supervisor will provide additional training until fidelity is achieved.

### Intervention delivery

Interventionists will be South African, female, and Black or mixed race to match AGYW and FC. Both interventions will be delivered over two sessions (about 10 h of content). We will enroll approximately 16 dyads per cohort, and we will enroll two cohorts every 6 weeks over 24 months. We will provide breakfast and lunch on both days. All intervention activities will occur at research sites in Klipfontein/Mitchells Plain.

#### Description of the interventions: Informed Motivated Aware and Responsible Adolescents and adults- South Africa (IMARA-SA)

The IMARA curriculum was subject to a comprehensive and systematic adaptation process prior to this RCT. The details of the adaptation process are presented elsewhere [81]. Separate FC and AGYW groups will run simultaneously, cover parallel content, and address individual, social, and structural drivers of HIV-risk (Table 1). Joint activities are designed to enhance FC credibility as a resource for HIV/STI prevention and facilitate practice of new communication skills. Additional activities will address conflict negotiation, assertive communication, and strengthening the AGYW-FC relationship. Interventionists will use interactive and experiential activities to address key constructs. The group format promotes structural change by building community norms for prevention (e.g., HTC and PrEP uptake) and reducing HIV stigma. IMARA-SA’s goals and motto emphasize strong FC-AGYW relationships, sisterhood, empowerment, and motivation for HIV/STI prevention, and build group cohesion. FC and AGYW will sign a pact to confirm commitment to the program. At the end of session 1, participants will receive homework to complete for session 2. Woven throughout IMARA-SA is the impact of mental health, gender-based violence, and alcohol and drug use on HIV-risk.

**Table 1:** IMARA-SA Curriculum

Theory	Selected IMARA-SA Activities
Individual Factors	
Knowledge, attitudes, beliefs, skills about HIV, STI, PrEP, HTC	Facts of HIV/AIDS/STI/PrEP/HTC; Prevalence for adolescent girls and young women; PrEP education video; Perceived vulnerability; Personal risk triggers (e.g., people, places, feelings) and risk plans; Demonstrate and practice condom use; De-mystifying HIV testing; Importance of regular HTC and PrEP where appropriate to risk behavior.
Mental health	Destigmatize mental health; Links between mental health problems and HIV-risk behavior; Recognize how feelings trigger risk taking; Teach healthy coping strategies; Recognize the value of the body; Substance use and risk taking; Referrals for counseling
Social Factors	
Family context Geographically constrained networks	Importance of strong FC-AGYW relationships, open and positive communication, and FC monitoring for AGYW mental health and reduced HIV/STI-risk; FC-AGYW warmth and positive interactions; Create personalized monitoring plans; Teach and practice effective communication with FC and family; Discuss risk in the context of neighborhood and sexual networks.
Structural Factors	
Gender dynamics Partner relationships HIV/AIDS stigma & discrimination	Expectations of women; Media images; Healthy and unhealthy relationships; intimate partner violence; Gender-role expectations and stereotypes; Impact of gender dynamics on HIV/STI risk; Implications of age discrepant partners and concurrent partnerships (e.g., the added importance of HTC and PrEP); Assertive communication with partners and peers; Changes in community norms to reduce stigma toward HIV/AIDS, PrEP, and HTC.

### Time- and attention-matched control: general health promotion

The HP control group is a time-matched family-based intervention previously delivered to families in SA and translated into Xhosa. The program promotes healthy living by encouraging good nutrition, exercise, and violence reduction (Table 2). Similar approaches will be used by interventionists, including interactive games, videos, worksheets, and group discussion. Like IMARA-SA, FC and AGYW will meet in separate groups and together to review material and learn about nutrition, substance use, exercise, and other relevant health topics. HIV/AIDS prevention is addressed briefly given the high prevalence in SA.

**Table 2:** Health promotion topics

Topics	Selected Activities
Media Literacy	Media influences on body image, weight, beauty; Obesity epidemic in SA and the role of the media (e.g., digital altering, manipulation)
Healthy Eating	Healthy eating; impact of fast food; Nutrition labels; Meal logs to track food choices
Violence	Types and prevalence of violence (gang, domestic, bullying, dating); Links between violence and health; Impact of families and communities; Tips to prevent violence.
Alcohol/Drugs	Prevalence of drug and alcohol use among teens; Impact of drugs and alcohol on the brain; Costs of drug/alcohol use on families and communities; Resources for counseling
Physical Activity	Impact of media and technology on physical activity (e.g., time spent on TV, phones); Benefits of exercise and relation to overall health and well-being; Practice and planning for one physical activity per day.

#### Contamination

We will minimize contamination across arms as follows. (1) Interventionists will not overlap, and training will occur separately. (2) Treatment conditions will occupy separate floors at the site to reduce participant contact. (3) We will assess exposure to IMARA-SA among control participants at 6- and 12-months. (4) We will adjust for exposure in the data analyses.

### Study assessments

AGYW and FC will complete 3-h baseline, 6-, and 12-month follow-up assessments, including STI testing and HTC. Surveys will be completed via ACASI. To facilitate retention, we will obtain extensive contact information for participants and three additional individuals who will always know how to reach them.

#### Primary outcomes

At 6-month follow-up, we will examine AGYW STI incidence (gonorrhea, chlamydia, and trichomoniasis), PrEP uptake and 1-month supply of medication, and HTC uptake.

Secondary Outcomes: At 12-month follow-up, we will examine AGYW STI incidence (gonorrhea, chlamydia, and trichomoniasis), PrEP prescription and 1-month supply of medication, and HTC uptake. At 6- and 12-month follow-up, we will assess AGYW sexual behavior (e.g., condom use, substance use during sex, number of partners, concurrent partners), using the AIDS Risk Behavior Assessment, [82] HIV incidence via HTC, and self-reported adherence to ART and PrEP (where appropriate) using Wilson’s 3-item scale [83]. We will use standard cost evaluation methods [84, 85] to assess IMARA-SA’s costs, including personnel time and non-personnel resources (e.g., travel expenses, snacks, incentives, curricular materials).

#### Mediators and moderators

We will assess HIV/STI knowledge, attitudes, beliefs, and skills, [86–88] mental health, trauma, and intimate partner violence exposure, [89–92] PrEP knowledge and attitudes, quality and quantity of FC-AGYW communication, [32, 93, 94] parental monitoring, [95] and warmth and closeness in the relationship, [96] gender dynamics and partner communication, [94, 97–99], HIV/AIDS and PrEP stigma, [100], peer influence, [82] and substance use [82]. At 6- and 12-month follow-up, control group participants will indicate if they heard about IMARA-SA (yes/no) and what they heard (open-ended) to assess contamination.

### Retention

We will collect participant contact information at baseline and the contact information for at least three individuals (i.e., collaterals) who will “always know where they are.” We will contact individuals monthly to update our information. We will use multiple methods, including text, email, or phone to reach them. One month before their 6- and 12-month follow-up, we will remind them of their next scheduled visit. For AGYW or FC whom we cannot reach, we will visit their homes and contact their collaterals for assistance. We will continue to seek them until the window for their assessment has closed.

Timeline

**Table:** 

Study Activities	Year 1				Year 2				Year 3			
Quarter	1	2	3	4	1	2	3	4	1	2	3	4
Preparation of tools/procedures, IRB approval	X	X										
Staff recruitment and training		X										
Baseline assessments and intervention delivery			X	X	X	X						
6-month follow-up assessments					X	X	X	X				
12-month follow-up assessments							X	X	X	X		
Data analyses, write-up, and results dissemination									X	X	X	X

### Data management

Each participant will be assigned a unique identification (ID) number, which will be used for survey and laboratory data. Most survey data will be collected using Qualtrics, a web-based platform maintained by the University of Illinois at Chicago (UIC). Data that are collected using paper forms will be double entered for accuracy. All paper data will be kept in locked cabinets at the research site. Data will be stored in password-protected cloud-based systems and secure servers maintained by UIC and DTHF. Laboratory data will be collected by trained clinic staff and recorded in participant case report forms, which will be stored under lock and key at the research site and routinely transcribed into an electronic spreadsheet. Data will be accessible only to study personnel. Co-PIs will review all external requests to use the data, and data files provided to individuals will be stripped of identifiers. Study investigators will check for the completeness and accuracy of data, and confidentiality of records will be upheld.

### Statistical analysis

We will generate frequencies and summary statistics of predictor and outcome variables to screen and clean the data. We will evaluate randomization by comparing intervention groups on baseline variables using chi-square tests for categorical variables and t-tests or nonparametric Wilcoxon rank-sum tests for continuous variables. In subsequent analyses, we will adjust for predictor variables on which baseline randomization is not successful. We will examine if AGYW who complete the 6- and 12-month assessments differ systematically on baseline data from those lost to follow-up. We will examine correlations among the variables, inspect scale reliabilities to create appropriate scales, and create summary scores as appropriate (e.g., mental health problems). We will use models with random intercepts to account for clustering (if needed) and test the significance of clustering of subjects within groups.

We will analyze binary outcomes using logistic regression and continuous outcomes using linear regression. We will test the effect of the intervention on the primary and secondary outcomes and theoretical mediators, adjusting for confounders and the outcome at baseline as additional independent variables. Analyses combining time points will use generalized linear mixed-models with subject-specific random effects to account for correlation within repeated measurements. We will test an average effect across time points and effects on patterns of change from 6- to 12-months using interactions with time. Survival analysis methods will assess the effect of the intervention on incident STIs, using extended Cox regression models with fixed and time-varying covariates to estimate univariable and multivariable hazard ratios for associations of exposures with incident STI. Treatment effects may differ systematically depending on various factors, such mental health or substance use. We will test mediator and moderator effects according to Baron & Kenny [101] and Cohen & Cohen [102]. We will include interactions between treatment condition and covariates in the GLM, and obtain estimates for regression coefficients of the interaction terms, 95% confidence intervals, and *p*-values for the hypothesis tests.

We will evaluate the CE of IMARA-SA with respect to STI acquisition, the net cost of the intervention to the health system adjusting for cost savings from STI reduction, and explore HIV incidence where possible. We will conduct individual CE analyses for each STI (i.e., gonorrhea, chlamydia, and trichomoniasis) to compare the CE of IMARA-SA to interventions that focus on only one STI, as is typical in the limited published literature on STI prevention. The main CE analysis will include all 3 STIs and assess the overall CE of IMARA-SA. In addition, we will conduct two supplementary analyses to estimate: (a) the CE of IMARA-SA when viewed strictly as an HIV prevention intervention; and (b) the CE with all 4 STIs (gonorrhea, chlamydia, trichomoniasis, and HIV). Finally, we will assess the net cost to the health system from implementing IMARA-SA, adjusting for cost savings related to the reduction of STIs. The main outcome for the analyses is the CE ratio, defined as the incremental cost of the program per infection prevented by the intervention.

### Sample size calculation

Power analyses determined the sample size needed to compare the effect of IMARA-SA and control interventions on AGYW HIV/STI incidence and PrEP and HTC uptake. Because binary outcome variables pose the greatest challenge for power, we evaluated power for STI incidence (yes/no)—our primary outcome—across a range of prevalence estimates over 12-months. All calculations used a 2-sided test with alpha = .05. Based on data in the Plus Pills study, [103] using a Cox proportional hazards model with a range of incidence estimates, we will have ≥80% power to detect hazard ratios ≤0.5 for STI incidence ranging from 20 to 30%, even with 25% attrition. Effect sizes for binary outcomes is given in terms of the relative risk (RR), where RR = 1 indicates no treatment effect. A RR < 1 indicates a protective effect of the intervention. With 80% retention, we will have ≥80% power to detect RRs ≤ 0.38 when the proportion in the control group is 20%, and RRs ≤ 0.65 when the proportion in the control group is 50%. We also calculated the standard effect size (d) in standard deviations for group mean comparisons with 80% power for continuous outcomes, where d = 0 is defined as no treatment effect. We will be able to detect a small to medium effect size (d = 0.36) even with 75% retention. Low HIV incidence among AGYW (2.54%) per year will allow us to explore incidence, but we do not expect sufficient statistical power to test intervention effects on HIV incidence.

### Data monitoring

Data will be monitored by Weststat on an annual basis. A designated monitor will visit the study site to review participant records, consent documents, source documentation, data collection instruments, screening/enrollment logs, visit checklists, baseline checklists, chart notes, referral forms, recruitment and outcome forms, participant evaluations forms, study blood draws, and survey data base entry. We will also assemble a Data Safety and Monitoring Board, which will review our activities to ensure participant safety and evaluate findings in an interim analysis of the data to determine if the RCT should continue or be stopped.

### Adverse event monitoring

The study team will monitor any potential adverse events or study harms. These will be documented and reported within 24 h to the study PIs and the IRBs.

### Dissemination plans

Findings will be disseminated through peer-reviewed publications, local and national presentations, and conferences, and to relevant stakeholder groups. The PIs will review and approve all materials prior to dissemination, including written reports and presentations. Data will be placed in a repository for public access once the study is complete and the primary outcome papers are published.

## Discussion

This study addresses a compelling need for innovative multilevel interventions to improve the HIV prevention and care continuum for AGYW and lays the foundation for sustainability. The ongoing disparity of new infections in SA AGYW [78] requires an expansion of prevention options that address individual, social, and structural drivers, [79] but research on evidence-based interventions with strong evaluation designs and CE is lacking for AGYW, especially those capitalizing on the strengths and assets of families. We describe herein the design of a RCT of a family-based HIV prevention program, IMARA-SA, to reduce incident STIs and improve uptake of HTC and PrEP among AGYW in South Africa. The intervention draws on a Social Personal Framework [48] which recognizes the multilevel influences of individual, social (family, partner), and structural (stigma, peer norms) characteristics on AGYW HIV-risk and prevention. We hope to extend the evidence base for family-based HIV prevention and uptake of PrEP and HTC among SA AGYW, a population at exceptionally high risk of HIV and STIs and for whom current advances in HIV prevention have not been realized. Importantly, the study responds to urging by the target population for family-based programming. Prior to funding, IMARA-SA was extensively vetted with key stakeholders and the DTHF community advisory boards for approval. This process was essential to ensure study uptake and acceptability broadly, and improve chances for sustainability.

We believe the study design has several strengths. We will leverage FC and the FC-AGYW relationship to support HTC and PrEP uptake and linkage to care (where appropriate). Although AGYW are the primary focus of the program, IMARA-SA targets both FC and AGYW to support one another in prevention efforts. The design offers a combination package that includes behavioral and biomedical intervention strategies and provides on-site access to HTC, PrEP, and STI treatment, eliminating typical barriers to service. Furthermore, we believe that IMARA-SA is responsive to the age, gender, and cultural needs of the population. Although originally designed and tested with African American 14–18-year-old girls in the US, our systematic approach to adaptation for the South African local context increases confidence in its local relevance. This study is among the first to evaluate the impact of a FC-AGYW intervention on HTC and PrEP uptake using a rigorous and culturally adapted trial of sufficient size to detect effects on AGYW STI incidence and explore HIV incidence. We consider it a potential innovation in the HTC and PrEP uptake environment, which given the smooth access to services, close collaboration with clinic partners, and brief intervention format (approximately 10 h), is well positioned for scale. IMARA-SA was designed to meet the standard for evidence-based interventions, advance intervention science with SA AGYW, and prepare for sustainability by engaging key stakeholders at the start and documenting the costs.

## Data Availability

A de-identified dataset will be made publicly available through the National Institute of Child Health and Human Development (NICHD) Data and Specimen Hub (DASH) https://dash.nichd.nih.gov.

## Abbreviations

AGYW: Adolescent Girls and Young Women
FC: Female Caregivers
SA: South Africa
ARBA: AIDS Risk Behavior Assessment
ACASI: Audio Computer Assisted Self Interview
CDC: Centers for Disease Control and Prevention
HIV: Human Immunodeficiency Virus
HTC: HIV Testing and Counseling
PrEP: Pre-Exposure Prophylaxis
RCT: Randomized Controlled Trial
US: United States
UIC: University of Illinois at Chicago
DTHF: Desmond Tutu Health Foundation
IMARA-SA: Informed, Motivated, Aware, and Responsible Adolescents and Adults- South Africa
HP: Health Promotion
STI: Sexually Transmitted Infection
